# Supplementary figures and images for: Identification of a Sub-Clinical Salmonella spp. Infection in a Dairy Cow Using a Commercially Available Stool Storage Kit
Source: Animals (Basel). 2023 Sep 4;13(17):2807. doi: 10.3390/ani13172807 (PMC10486393; doi:10.3390/ani13172807)

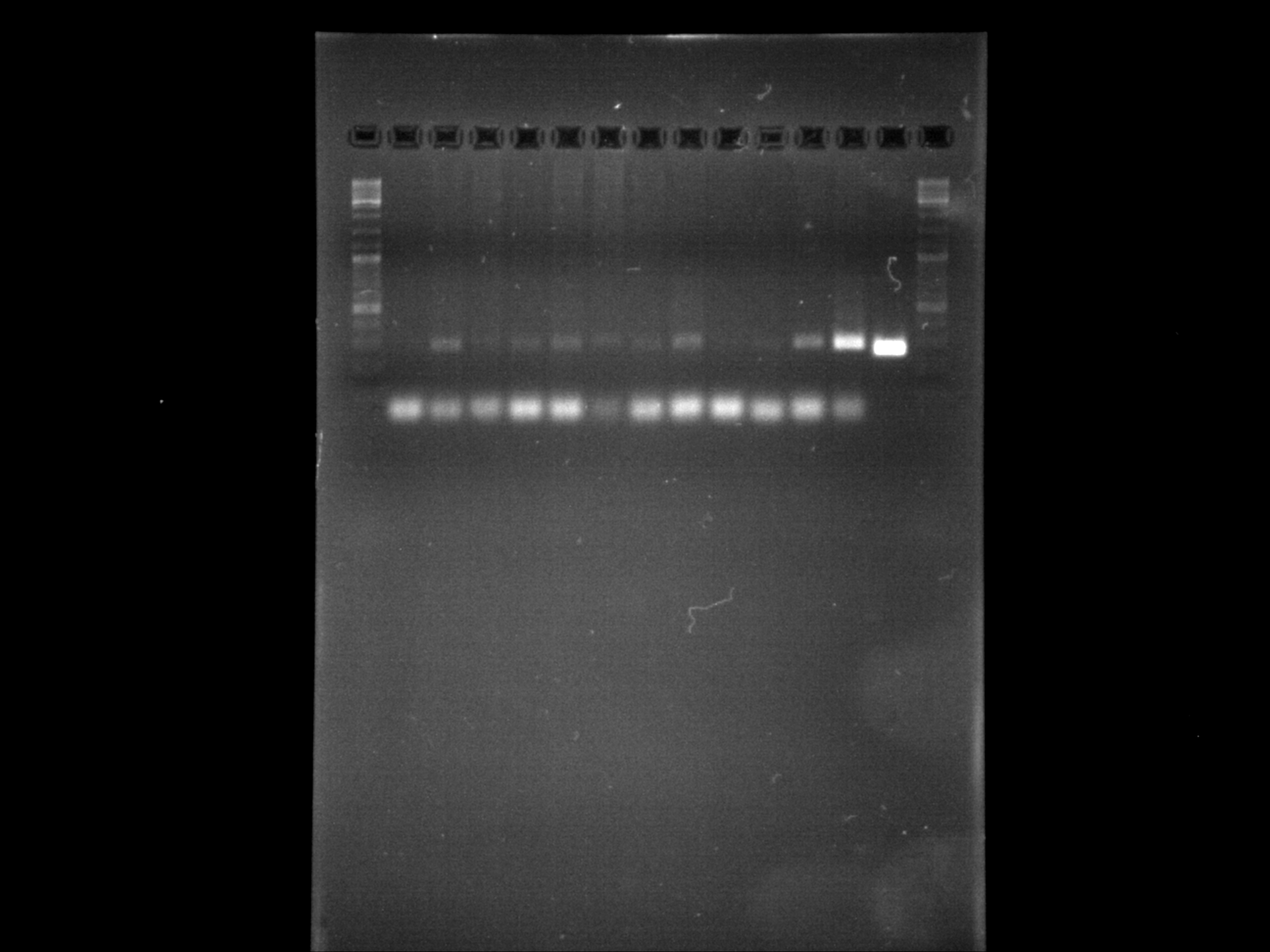

Supplement: Supplementary file 1 [file animals-13-02807-s001.zip › Figure 2 original.tif]

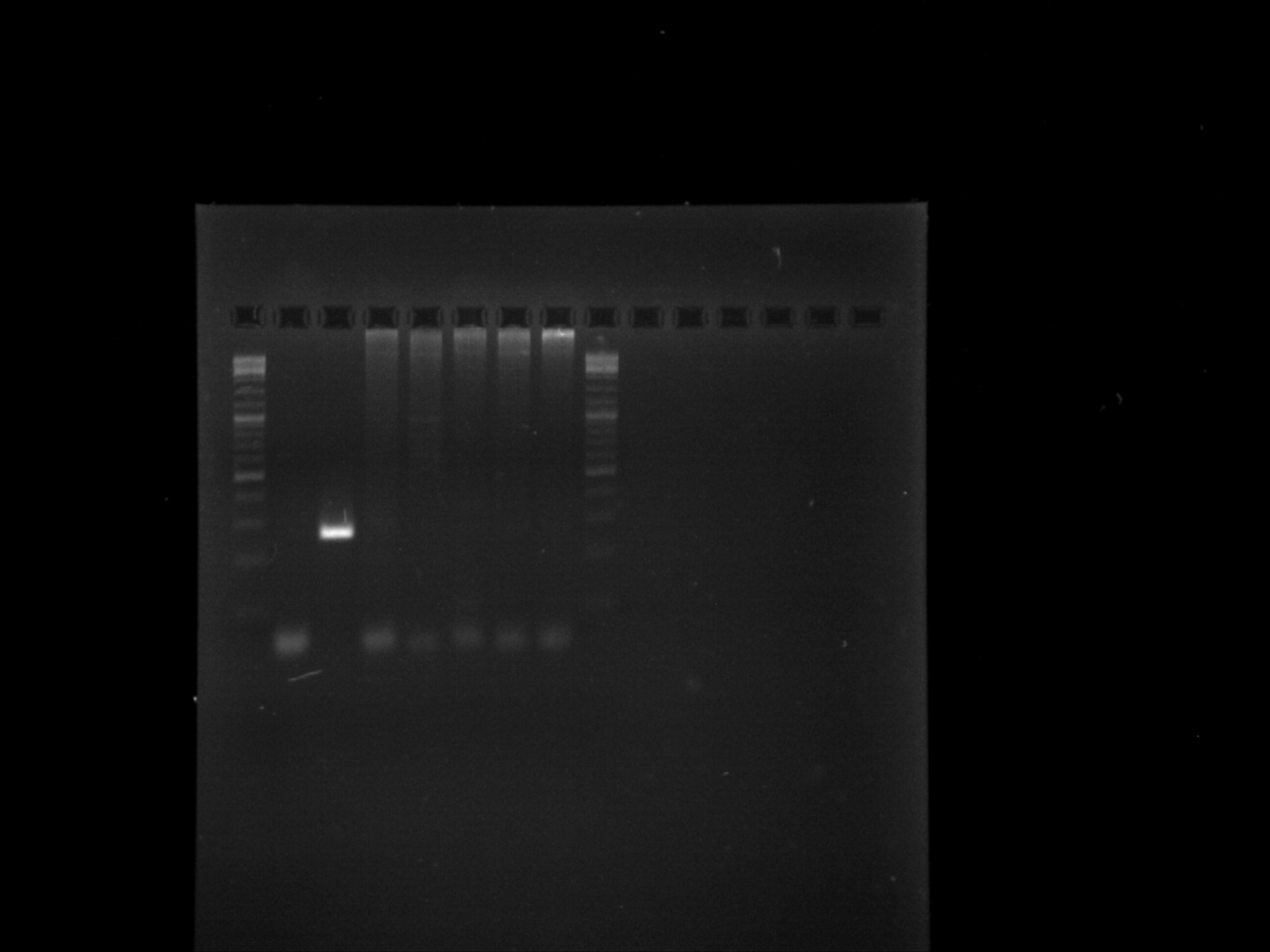

Supplement: Supplementary file 1 [file animals-13-02807-s001.zip › Figure A1 original.jpg]

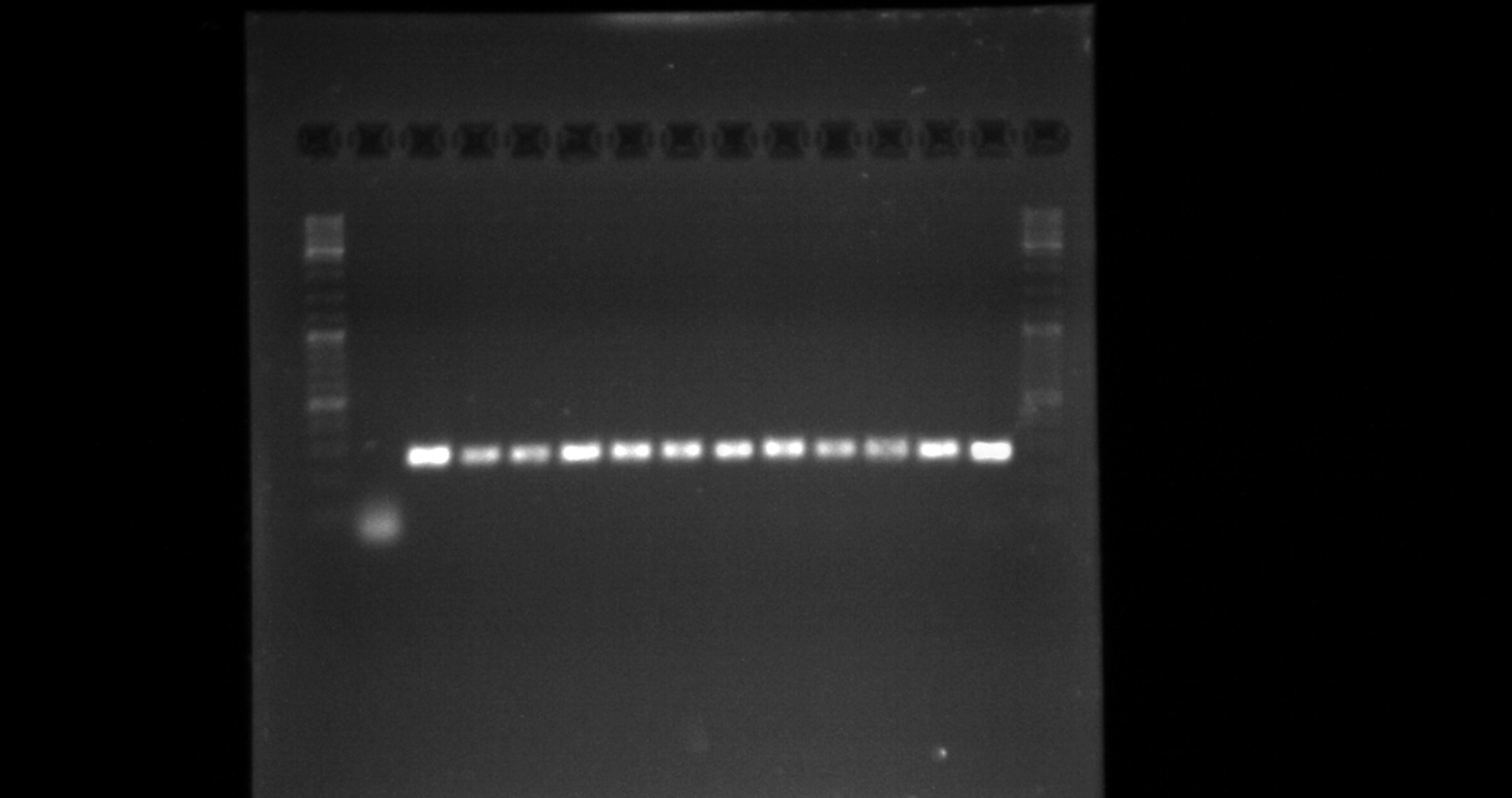

Supplement: Supplementary file 1 [file animals-13-02807-s001.zip › Figure A2 original.jpg]

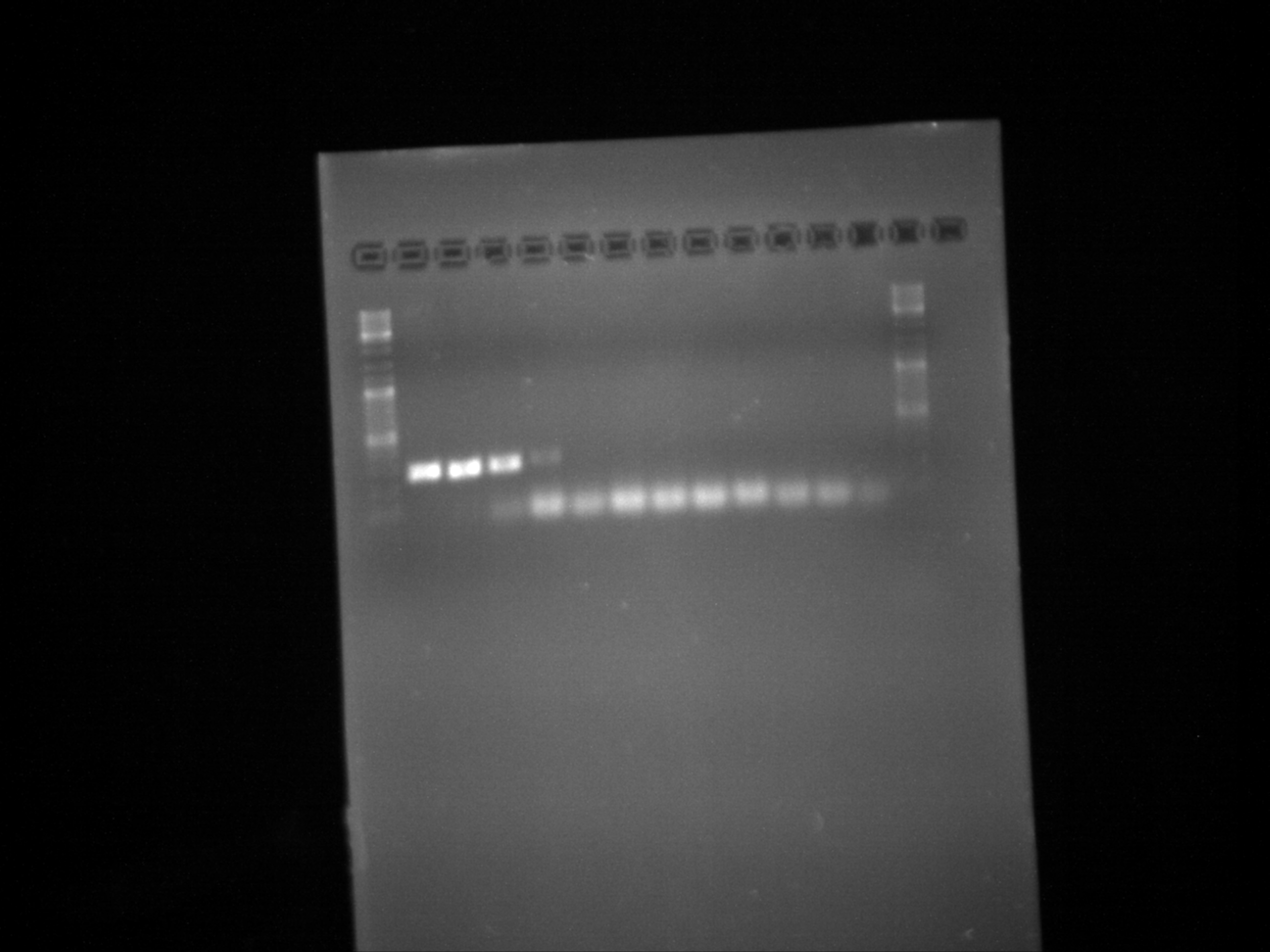

Supplement: Supplementary file 1 [file animals-13-02807-s001.zip › Figure A3 original.tif]
